# Direct observations of microbial community succession on sinking marine particles

**DOI:** 10.1093/ismejo/wrad010

**Published:** 2024-01-10

**Authors:** Brandon M Stephens, Colleen A Durkin, Garrett Sharpe, Trang T H Nguyen, Justine Albers, Margaret L Estapa, Deborah K Steinberg, Naomi M Levine, Scott M Gifford, Craig A Carlson, Philip W Boyd, Alyson E Santoro

**Affiliations:** Department of Ecology, Evolution and Marine Biology, Marine Science Institute, University of California, Santa Barbara, CA 93106, United States; Present address: Institute of Oceanography, National Taiwan University, Taipei 106, Taiwan; Monterey Bay Aquarium Research Institute, Moss Landing, CA 95039, United States; Department of Earth, Marine, and Environmental Sciences, University of North Carolina at Chapel Hill, Chapel Hill, NC 27599, United States; Department of Biological Sciences, University of Southern California, Los Angeles, CA 90089, United States; Department of Integrated Sciences, Fulbright University Vietnam, Ho Chi Minh City 756000, Vietnam; Department of Ecology, Evolution and Marine Biology, Marine Science Institute, University of California, Santa Barbara, CA 93106, United States; School of Marine Sciences, Darling Marine Center, University of Maine, Walpole, ME 04573, United States; Coastal & Ocean Processes Section, Virginia Institute of Marine Science, William & Mary, Gloucester Point, VA 23062, United States; Department of Biological Sciences, University of Southern California, Los Angeles, CA 90089, United States; Department of Earth, Marine, and Environmental Sciences, University of North Carolina at Chapel Hill, Chapel Hill, NC 27599, United States; Department of Ecology, Evolution and Marine Biology, Marine Science Institute, University of California, Santa Barbara, CA 93106, United States; Institute for Marine and Antarctic Studies, University of Tasmania, Hobart, Tasmania 7001, Australia; Department of Ecology, Evolution and Marine Biology, Marine Science Institute, University of California, Santa Barbara, CA 93106, United States

**Keywords:** 16S rRNA, metagenomes, sinking particles, particle lability, community succession, island biogeography, bacterial community diversity, individual particles, carbon export

## Abstract

Microbial community dynamics on sinking particles control the amount of carbon that reaches the deep ocean and the length of time that carbon is stored, with potentially profound impacts on Earth’s climate. A mechanistic understanding of the controls on sinking particle distributions has been hindered by limited depth- and time-resolved sampling and methods that cannot distinguish individual particles. Here, we analyze microbial communities on nearly 400 individual sinking particles in conjunction with more conventional composite particle samples to determine how particle colonization and community assembly might control carbon sequestration in the deep ocean. We observed community succession with corresponding changes in microbial metabolic potential on the larger sinking particles transporting a significant fraction of carbon to the deep sea. Microbial community richness decreased as particles aged and sank; however, richness increased with particle size and the attenuation of carbon export. This suggests that the theory of island biogeography applies to sinking marine particles. Changes in POC flux attenuation with time and microbial community composition with depth were reproduced in a mechanistic ecosystem model that reflected a range of POC labilities and microbial growth rates. Our results highlight microbial community dynamics and processes on individual sinking particles, the isolation of which is necessary to improve mechanistic models of ocean carbon uptake.

## Introduction

The ocean’s biological carbon pump removes recently fixed carbon dioxide (CO_2_) from surface ocean ecosystems and sequesters a proportion of it in the deep ocean through the production and export of sinking particles [1]. The combined activities of zooplankton, phytoplankton, and other microbes control the relative strength of this downward pump and, therefore, its role in mediating atmospheric CO_2_ levels over short- and long-time scales [2]. Decomposition by particle-attached heterotrophic bacteria and archaea is a key process influencing how much surface-produced carbon sinks through the mesopelagic and away from the atmosphere (i.e. transfer efficiency) [3-5]. Whether or not specific microbes, assemblages, and their metabolic capabilities control the transfer efficiency of particles into the deep ocean remains an open question [6].

The growth rate of particle-attached bacteria is modeled as one of the most influential factors controlling microbially mediated sinking particle remineralization rates in the ocean [7]. For rapid growth to occur, the particle-attached community must reach a critical population density threshold [8, 9], and sinking particles require an efficient exchange of solutes [10], offering potential explanations for the “particle decomposition paradox,” whereby microbial respiration on particles is thought to be low despite high particle attenuation with depth [11]. Various processes can limit net microbial growth on particles, including mortality, the microbial community’s metabolic potential, and the sinking particles’ organic composition [8, 9, 12]. As particles sink and are remineralized by the particle-attached community, the metabolic capabilities required to survive on that particle should also change. Datta *et al*. [13] observed microbial community succession on model chitin particles through changes in overall richness and shifting physiological capabilities. These models and observations indicate that changes in microbial structure are an important driver of sinking particle remineralization and the depth of carbon sequestration in the ocean. However, determining whether these microbial community dynamics occur in natural ecosystems is hindered by insufficient methodologies, limiting the number of observations documenting this process [14, 15].

The most common methods for analyzing particle-attached microbes in the natural environment obscure the dynamics occurring on individual particles. Prior studies have primarily used size-fractionated filtration of water column samples to identify the differences between particle-attached and free-living microbial communities. This is subject to a range of biases, including filtration volume and particle disaggregation during sampling [16]. Moreover, it is unclear whether size-fractionated filtration is representative of material sinking through the water column and contributing to downward carbon export. Targeted collection of sinking particles and their associated communities has been accomplished using marine snow catchers (MSCs) [17, 18] and sediment traps, which collect sinking particles that are retained within a dense sample preservative [6, 19]. Sediment traps can collect a range of fast and slowly sinking particles either in bulk or embedded in polyacrylamide gel layers, the latter of which allows for single particle isolation and has only been used in microbial community composition studies in limited locations to date [20-22]. Among the various sinking particle collection methods, studies consistently find that bacterial communities associated with particulate matter differ from free-living communities in metabolic capacity, having a greater number of polysaccharide degradation and amino acid transport genes, among other metabolic capabilities [6, 23, 24]. Taxonomic and metabolic differences have been observed in communities associated with particles using these sampling approaches [25-28], but the identified metabolisms are also likely influenced by the sample collection method.

We collected 578 observations of microbial communities on marine particulate matter to determine (i) to what extent sample collection method and environmental conditions influence community composition, (ii) if changes in particle-attached community composition are associated with the transfer efficiency of particulate carbon into the deep ocean, and (iii) if theoretical predictions of particle-attached microbial community succession could be observed in a natural ocean ecosystem. Samples were collected during a 24-day cruise from surface depths to 500 m near the iron-limited, small algal cell-dominated Ocean Station Papa in the subarctic North Pacific Ocean [29-31]. Our analysis includes 377 individually isolated sinking particles, revealing a more nuanced view of microbial community dynamics on individual particles as compared to bulk collection methods. In addition, metagenome-assembled genomes of the dominant particle-attached taxa within individual particles indicate differences in metabolic capabilities associated with the attenuation of sinking particulate organic carbon flux over depth and time.

## Methods

### Cruise framework

Samples for this study were collected aboard R/V *Roger Revelle* cruise RR1813 as part of the EXport Processes in the Ocean from RemoTe Sensing (EXPORTS) program in the sub-Arctic North Pacific near Ocean Station Papa (50.1°N, 144.9°W) between 15 August and 7 September 2018, at depths of 95, 110, 145, 195, 330, and 500 m. Cruise RR1813 was conducted as a Lagrangian, “process”-based study, tracking coherent upper ocean water masses over time. Drifting, neutrally buoyant, surface-tethered sediment traps, and MSCs were deployed during three 8-day “Epochs” during the cruise. A 24-bottle CTD-mounted rosette containing 24 Niskin bottles was deployed daily to collect nutrients, chlorophyll, and other biogeochemical samples [29]. The rates of net primary production (NPP), gross carbon and secondary production, and euphotic zone 16S rRNA gene amplicons for the 0.2 μm filters were recently published [32].

### Sample collection

Six unique sample types were collected in the dataset presented here: (i) individual sinking particles captured in polyacrylamide gel traps, (ii) bulk particles from drifting sediment traps, (iii) fresh zooplankton fecal pellets, (iv) particles collected using MSCs, and (v) suspended particles collected onto 5.0 μm pore-size filters, as well as (vi) the free-living microbial community as captured on 0.2 μm pore-size filters (Supplementary Fig. 1).

Sinking particle samples were collected from two different sediment trap designs deployed for 3–6 days, as previously detailed [33]. A surface-tethered trap array collected particles at 95, 145, 195, 330, and 500 m depths, and neutrally buoyant sediment traps were deployed at 95, 145, 195, and 330 m depths. Particles were collected from polycarbonate tubes, each with a collection area of 0.0113 m^2^. Sinking fluxes are from Estapa *et al*. [33]. Bulk particles for molecular analysis were collected from tubes on the surface-tethered array only, containing an ammonium sulfate-based preservation solution similar to RNALater [34] to preserve genetic material. Upon recovery, sediment trap tubes were left to settle for 1 h, after which the overlying seawater was siphoned off. Samples were then vacuum-filtered onto 47 mm, 0.2 μm pore-size polyethersulfone (Pall Supor) filters, flash frozen, and stored at −80°C until processed in a shore-based lab. Individual particles were collected from tubes containing polyacrylamide gel overlain with filtered (<0.2 μm) seawater [35, 36]. Prior to particle removal, each particle was imaged using a stereomicroscope (SZX16, Olympus, Tokyo, Japan), and images were later used to measure particle size. Individual particles >100 μm were removed from the gel using a Gilson P1000 pipettor after first filling the pipette tip with ~200–300 μl nuclease-free water. Particles were then transferred to cryovials, flash-frozen, and stored at −80°C until further processing in the lab.

Samples for DNA analysis were also collected from MSCs with nominal 90 L volumes following procedures outlined in previous studies [18, 37]. MSCs were deployed three times per Epoch at three unique depths for a total of nine deployments. After deployment and recovery, the seawater within the MSC sat on the deck for ~2 h, whereafter three unique DNA samples were collected. One sample was poured out of a spigot near the top of the MSC and so is considered representative of “non-sinking” particles. Another sample was poured out of a spigot near the base of the MSC and is considered representative of the “slowly sinking” particles, but this sample also contained water from the nonsinking fraction. The final sample was collected from the bottom tray of the MSC from a polycarbonate tray containing “fast-sinking” particles but also contained water from the nonsinking and slowly sinking particles. A 1 l volume for each fraction was vacuum-filtered onto 47 mm 0.2 μm polyethersulfone filters, flash-frozen, and stored at −80°C until processed further in the lab.

Fresh fecal pellets from salps (*Salpa aspera*) and hyperiid amphipods (*Vibilia propinqua*) were collected by vertical plankton net tows within the top 0–100 m deployed at night [38, 39]. Animals were placed in surface seawater, and fecal pellets were collected using wide-bore pipettes from the bottom of the containers. Salp fecal pellets (*n* = 7) were isolated during Epoch 1, and hyperiid amphipod (*V. propinqua*) fecal pellets (*n* = 5) were isolated during Epoch 2.

Six times throughout the cruise, 2 l samples from the water column were collected from Niskin bottles deployed on a rosette sampler and pressure filtered using a peristaltic pump onto inline 25 mm, 5.0 μm pore-size polyester (Sterlitech), and 0.2 μm pore-size polyethersulfone (Pall Supor) filters housed in Swinnex filter holders, at depth of 95, 145, 195, 330, and 500 m depths. Separately, at an additional six times during the cruise, 1 l samples were pumped using positive pressure onto 0.2 μm Sterivex filter cartridges from 5, 20, 35, 50, 65, 80, 95, 120, 145, 195, 330, and 500 m depths. The microbial communities, representing the “free-living” community, from these samples are previously reported [32]. Filters were flash-frozen and stored at −80°C until further processing in the lab.

### 16S small subunit ribosomal RNA gene amplification and analysis

Bulk trap-collected particle, MSC, and the 5.0 μm and subeuphotic zone 0.2 μm filters were extracted using a PowerViral DNA/RNA extraction kit (Qiagen, Hilden, Germany). Euphotic zone 0.2 μm filters were extracted using the phenol:isoamyl:chloroform method [40]. Individual particles collected from gels were first thawed and then extracted using a 5%–10% solution of Chelex 100 resin (BioRad, Hercules, CA, USA) in nuclease-free water (0.1 g ml^−1^). Particles within Chelex were vortexed for 2 min, then incubated in a water bath at 95°C for 10 min, followed by a second round of vortexing and incubation. Samples were then centrifuged, and the top fraction containing nucleic acids was removed for further purification. The DNA within the liquid was concentrated using the Genomic DNA Clean and Concentrator-10 (Zymo Research, Irvine, CA, USA) to a final elution volume of 50 μl.

Extracted DNA samples were barcoded during polymerase chain reaction (PCR) amplification using custom V4 515F-Y (5′-GTGYCAGCMGCCGCGGTAA-3′) and 806RB (5′- -3′) primers with custom adapters [41-43]. Amplified and gel-purified libraries were sequenced by a MiSeq (Illumina) at the University of California Davis Genome Center. Sequencing reads were trimmed and assigned to taxonomies based on a DADA2 pipeline [44] using matches to the SILVA SSU / LSU 138 database (accessed in December 2021). After low-read samples were removed (< 500 reads), 7965 total amplicon sequence variants (ASVs) were detected across 578 samples.

Rare taxa were not the focus of the current study, so ASVs with fewer than 20 reads in fewer than two samples were removed, resulting in 3981 ASVs. A comparison of sample distributions based on ordination, richness, and diversity metrics did not noticeably differ between the trimmed set of ASVs (*n* = 3981) and the complete set of detected ASVs (*n* = 7965). Read depths for the trimmed sample set ranged from 6199 to 113 934 reads and averaged 36 629 ± 16 565 per sample. The number of reads per sample was not influenced by the various sample collection types, where the mean for each sample type fell within the mean and standard deviation of the total sample set, so samples were not rarefied [45] prior to comparison methods. Additionally, rarefaction curves indicated that the number of unique ASVs plateaued by ~5000 reads (Supplementary Fig. 2), suggesting that the maximum number of taxa were sampled across the various sample types presented here.

### Data analysis of 16S small subunit ribosomal RNA gene-based communities

For sample comparisons (*n* = 578), total reads (out of the trimmed 3981 ASVs) were converted to relative abundances and then square root-transformed for further analysis to stabilize the sample variance. An analysis of similarity test (ANOSIM, using R function “anosim”) [46, 47] was used to test differences based on sample collection type when comparing bulk sediment trap particles, individual particles, MSC samples, 5.0 μm filters, and 0.2 μm filters. Then, Bray–Curtis dissimilarities were compared via clustering (“hclust” function with Ward.D2 clustering option in R) and a nonmetric multidimensional scaling (NMDS) ordination. Samples were best separated into eight clusters using the “clusGap” function in R based on 500 Monte Carlo bootstrap samples and the smallest number of clusters, given the set distance of standard errors away from the first local maximum [48]. NMDS stress values converged on a value of 0.08 (after 28 iterations and k = 3), indicating that the predicted sample distances were sufficient for meaningful interpretation of sample ordination placements relative to one another. The Bray–Curtis-based dissimilarity clustering and ordination results were compared with weighted UniFrac distances using a rooted phylogenetic tree [49], and it was found that samples were similarly separated based on the collection type method except that more of the bulk particles (*n* = 19) clustered separately from all other sample types by weighted UniFrac distances. ASV indicators were determined for each of the six sample types identified here [50].

ASV richness and alpha diversity (Shannon index (H)) metrics were determined on the trimmed sample set using PAST software (v3.0). Patterns of diversity by depth and sample type were similar to richness, so only richness is presented in the main text.

### Sinking flux model

The modeled ecosystem particulate organic carbon (POC) fluxes, microbial community composition, and POC consumption rates were calculated using a previously developed mechanistic model [8]. This model describes the micro-scale dynamics on organic particulates as they sink through the water column. The model represents a range of POC lability types where particle lability is defined as the degradation rate of a specific POC type (i.e. chemical composition) by a specific microbial group (i.e. with specific enzyme kinetics). High particle lability refers to a high degradation rate of the POC and vice versa, and POC lability is assumed to be log-normally distributed [8]. Bacterial population dynamics on each particle were defined as a function of bacterial detachment rate, mortality rate, encounter rate, and maximal growth rates as previously described [8]. Two maximal growth rates were used, where a rate of 7.2 day^−1^ represented fast growers, such as *Pseudoalteromonas* [51] and *Psychromonas psych6C06* [9], and 1.2 day^−1^ represented slower growers [52]. Although the maximum growth rates presented here are relatively high for marine bacteria, previous studies have identified elevated bacterial growth rates of up to 15 day^−1^ for free-living bacteria [53, 54] and of up to 16 day^−1^ for particle-attached bacteria [55] and up to 35 day^−1^ under enriching conditions [56, 57]. The temperature scaling on growth rate was validated against experimental data for marine heterotrophs.

Two types of model simulations were conducted, as previously described [8]. First, observed water column POC flux measurements were compared against model simulations using the complete particle size spectra (particle size distribution slope equal to −3). Specifically, the model was initialized with 69 000 particles at 100 m with particle sizes ranging from 50 to 4000 $\mathrm{\mu}$m in diameter, particle lability from 10 to 1000 mmol C_POC_ mmol C_cell_^−1^ day^−1^ (i.e. removal of POC by attached bacterial per day, where C_POC_ represents a sinking particle and C_cell_ represents the community of attached bacteria), initial cell density from 400 to 2800 cells mm^−2^, maximal growth rate of particle-associated bacteria from 1.2 to 7.2 day^−1^, and a density of free-living bacteria from 10 to 500 cell mm^−3^. Four average particle labilities were tested with particles initialized using a lognormal range around the average value (ranging from low to high lability): 50, 100, 200, and 500 mmol C_POC_ mmol C_cell_^−1^ day^−1^. Given that the POC flux at 95 m was variable over the cruise, and to compare the changes in the POC flux between the model and field observations, the fraction of POC flux was standardized as the ratio between the POC flux at depth to the POC flux at the model initialization depth (100 m). We also estimated mean particle consumption rates in the model as previously described [8] and compared these against the observed rates.

As a second type of model simulation, to compare with the observed change of bacterial community on salp pellet particles with depth, model simulations were conducted using only particles with 2000 and 4000 μm in diameter at formation, slightly larger than the observed salp diameters in gel traps. These large particles were initialized with the same range of microbial growth rates, encounter rates, and initial microbial population. We then calculated the average relative abundance of each modeled bacterial type from 100 m to 500 m.

### Estimated turnover times for particle-attached *Moritellaceae*

To provide an observation-based estimate of growth rates used in the model, we estimated the growth rates for a taxon that appears at depth using some basic assumptions about particle size, cell abundances, and particle sinking rates. We start from a mean particle diameter of 600 μm or surface area of 1.13 × 10^6^ μm, assuming the particle is a sphere. If at least 50% of the sphere is occupied, and the average diameter of a bacteria is 0.4 μm, this would result in about 1 × 10^6^ bacterial cells per particle. Then, using the increases in the relative abundance of *Moritellaceae* with depth, this would predict a cell abundance increase from 2.8 × 10^5^ cells per particle at 95 m to 1.3 × 10^6^ cells per particle at 500 m. A particle sinking velocity of 200 m day^−1^ with this population change would result in a turnover of 0.7 day^−1^. Had the particle been 1200 μm in ESD and the sinking velocity was 500 m day^−1^, this would result in a turnover of 1.9 day^−1^.

### Metagenomic sequencing and analysis

Extracted DNA from 25 individual particles was pooled from five unique time points and/or depths, and the DNA was concentrated using Amicon Ultra Centrifugal filters (3 kDa; Fisher Scientific). Four bulk sediment trap particle samples were also included and did not require a concentration step. Samples were processed on a NextSeq 500 platform (Illumina), producing 150 base pair paired-end reads for metagenomic shotgun sequencing at UCSB’s Biological Nanostructures Laboratory. Quality control and adaptor removal were performed with Trimmomatic56. The metagenomes were annotated using a DIAMOND-based [58] search against the NCBI Refseq protein database, and the MAGs were assembled using metaSPAdes [59]. Contigs were binned using MaxBin and CONCOCT [60, 61], then consolidated using DAS Tool and CheckM [62, 63]. Protein-coding genes for each bin were identified and annotated using Prokka [64] and SignalP 6.0 [65]. Carbohydrate enzyme KEGG orthologies (KOs)-associated modules were annotated using METABOLIC C [66].

## Results and discussion

### Environmental context

Over the month-long station occupation, previous evidence demonstrated that there was an increase in POC flux at 95 m and attenuation during the third (out of three) trap deployment; each deployment is referred to as an “Epoch” [33]. This was also associated with early Epoch 3 increases in surface ocean NPP, nitrate-based production, chlorophyll-a, suspended POC, bacterial biomass, and secondary production (as bacterial carbon demand; BCD), the latter of which was influenced by the production of relatively more labile dissolved organic matter (Supplementary Fig. 3) [32, 67]. Temperatures ranged from 14.1°C at 5 m to 3.9°C at 500 m (Supplementary Fig. 4) and exhibited minimal temporal variability over the cruise [29].

### Depth and particle size drive dynamics

**Figure 1:**
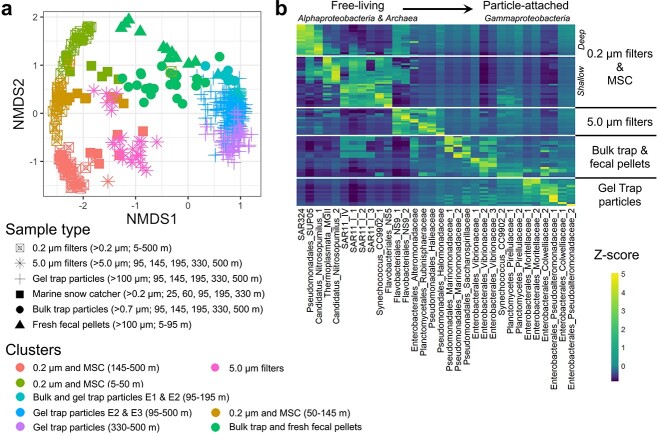
Sample collection type influences associated microbial communities; (A) Bray–Curtis dissimilarity-based nonparametric multidimensional scaling ordination of 16S rRNA ASVs separate by sample collection type and Ward.D2 clusters (see Supplementary Fig. 5 for clustering); (B) heatmap of indicator ASVs by sample collection type, represented as *z*-score normalized values based on relative abundances for all samples; noted in the *x*-axis of (B) are the order and family names for indicator ASVs that progress from *Alphaproteobacteria* to *Gammaproteobacteria*; fecal pellets = freshly collected; “E” = Epoch.

Based on amplicon sequencing of the 16S small subunit ribosomal RNA gene (16S rRNA), microbial community composition significantly differed (ANOSIM *r* = 0.93 and *P* = .001) among the six sampling methods (Fig. 1 and Supplementary Fig. 5). Bulk sediment trap samples, 5.0 μm filter samples, and particles in MSCs on average likely represent smaller, more slowly sinking particles compared with individual particles isolated from gels. Depending on how samples were isolated, these methods could also capture a combination of the free-living and particle-associated communities, as in the MSC’s tray fraction, which ordinated closer to bulk particle assemblages than the free-living community (Fig. 1).

**Figure 2:**
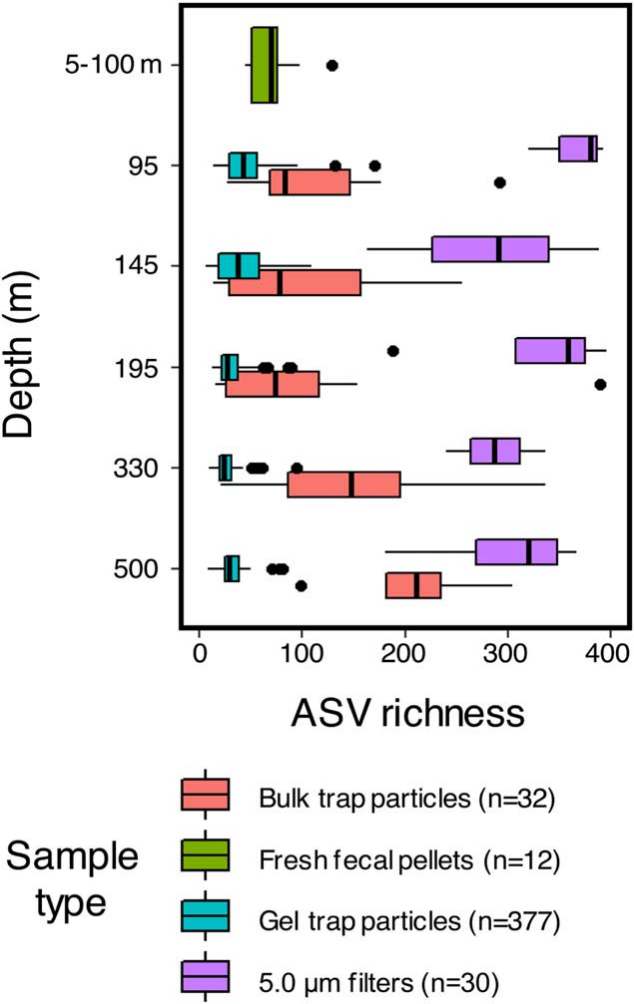
Communities on individual particles differ by collection method; ASV richness differs by particle collection type (i.e. 5.0 μm filters, individual particles from gel traps, bulk particles from sediment traps, fresh zooplankton fecal pellets); the line inside the box plots represents the median ASV richness, and the whiskers represent the minimum and maximum values excluding outliers (1.5 times the interquartile range, black dots).

Differences among sample methods were also observed with depth (Fig. 2). Depth is a primary driver of the free-living bacterial community composition over the water column (0–~4000 m) of the oceans [68-70] and could be a factor setting the community composition on particles [71]. Microbial community richness also significantly differed across the modes of particle sample collection (two-tailed *t*-tests, *P* < 0 .05) (Fig. 2), decreasing from >5.0 μm particles to the bulk trap particle assemblage to fresh fecal pellets to individual particles. The bulk sediment trap-collected particle communities doubled in richness with depth between 145 and 500 m, whereas the richness of individual particle communities decreased by half (Fig. 2). Decreasing richness with depth on the individual particles differs from the relatively minimal change in richness with depth for communities observed on 5.0 μm filters, which previously has been hypothesized to reflect a stronger vertical connectedness in such samples [72, 73]. Other recent evidence suggests that particle-associated bacterial communities may also share relatively more similarities in the subsurface across latitudes in the Pacific Ocean [74]. The differing trends in community richness with depth among methods (Fig. 2) suggest that colonization and community assembly patterns differ depending on how particles became modified (e.g. by biota or disaggregated) as they settle through the water column [15, 36, 75].

Prior studies demonstrate that microbial communities associated with sinking particles can originate directly from the guts of zooplankton [20], which were also the largest source of sinking particles during the EXPORTS expedition through fecal pellet production [36]. Fecal pellets freshly isolated by incubating live zooplankton collected from net tows (5–95 m) in particle-free seawater had an ASV richness that was low relative to the bulk trap assemblage and only slightly higher than the various types of individual particles (e.g. pellets, detritus) collected from the shallowest trap depth at 95 m (Fig. 2). Thus, the freshly produced fecal pellets entered the water column with a relatively low species richness that continued to decrease as the larger individual particles sank. The significantly reduced ASV richness associated with individual particles from gel traps and fresh fecal pellets compared with other collection methods demonstrates the importance of distinguishing among modes of particle collection and highlights a potential taxa selection mechanism (e.g. gut microbiota as a source, followed by successional patterns) that persists throughout the upper mesopelagic zone. The pattern of low and decreasing ASV richness observed on larger (e.g. >100 μm) sinking particles collected from gel traps would be otherwise obfuscated (i.e. “contaminated”) by particle-associated communities collected by other means that combine particles of varying size, age, and origin, as indicated by elevated richness (Fig. 2). A subset of sinking particle ASVs spanning domains and orders commonly associated with free-living communities were detected in all sample types (Fig. 1B and Supplementary Fig. 6), suggesting either that the free-living taxa colonized particles or that particles were significant contributors to the free-living community at depth [76]. Overlapping free-living and particle-associated ASVs included members of *Pseudoalteromonadaceae*, *Alteromonadaceae*, *Vibrionaceae*, *Cellvibrionaceae*, *Pirellulaceae*, and *Cyanobiaceae* (Supplementary Table 1).

The depth-related patterns we observed in microbial community richness provide evidence that successional dynamics occur on sinking particles in the open ocean. In the laboratory, particle-associated microbial communities on model chitin particles transitioned from an initial high-diversity attachment phase to a low-diversity “selection phase” over time [8, 13]. Here, we also observed a decrease in microbial community richness with depth on most individual particle types (Fig. 2). With typical sinking speeds of ~50–100 m day^−1^ for particles with equivalent spherical diameters (ESDs) ranging 100–600 μm [77], trap-intercepted particles would be collected 1–2 days after their production in the surface ocean, within the selection phase that was associated with relatively low diversity proposed by [13]. The trend of decreasing diversity with depth was not observed in methods capturing the bulk particle assemblage (MSC and bulk sediment trap), and so those particle types may have been replenished by particles in the earlier stages of colonization, again emphasizing the power of isolating individual particles for understanding microbial community dynamics.

**Figure 3:**
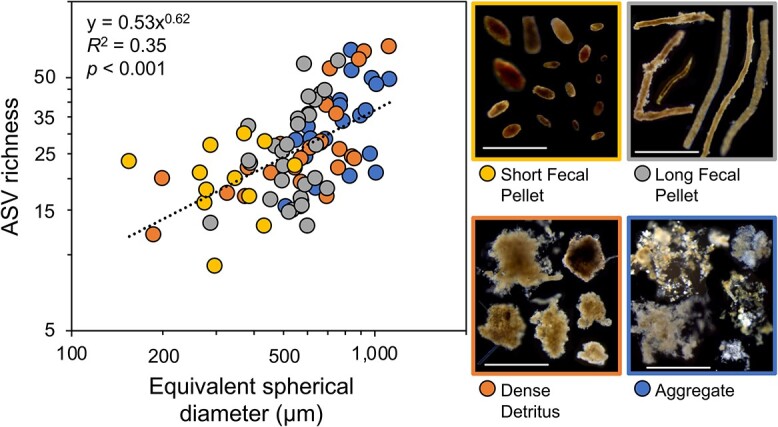
Communities on individual particles differ by particle size; ASV richness was significantly correlated (*P* < 0 .001) with the ESD of individual particles; scale bars in images: 1000 μm.

Individual sinking particles collected from gels within sediment traps also uniquely identify associated patterns in microbial community assembly by particle type and size (Fig. 3) [21]. Particle size is of particular importance because it may influence particle colonization rates and the availability of ecological niches [78, 79]. This is analogous to findings from terrestrial ecology, where plant and animal species richness has been shown to increase with island size [80]. Bacterial community richness has been shown to increase as a power law relationship with habitat size [78, 79, 81-84] and particle size classes from 0.2 to 200 μm throughout marine water columns [72, 85]. Our data indicate that this is also the case for particle-attached bacterial communities in the ocean. We observed a significant positive correlation (*R*^2^ = 0.35, *P* < .001, *n* = 89) between ASV richness and the ESD of individual particles (Fig. 3), suggesting that the theory of island biogeography could apply to sinking particles. The diversity of bacterial communities associated with marine particles, and aggregates in particular, exhibits a relatively steep species-area relationship (slopes of 0.62 in Fig. 3 and 0.4 to 0.6 in Lyons *et al*. [78]) when compared with larger island environments and larger organisms (e.g. slope of 0.05 to 0.3 in Bell *et al*. [82]). This further implies that larger particles have higher colonization rates irrespective of encounter rates [86], offering protected microhabitats that would thereby allow more diverse bacterial populations to reach critical density thresholds, as demonstrated by models [8]. However, due to the fractal nature of the dense detritus and aggregates [87], a larger surface area is available than is represented by the particle ESD. Therefore, the resulting underestimation of those particle habitat sizes using ESD could have also led to the steeper slope observed here (Fig. 3).

**Figure 4:**
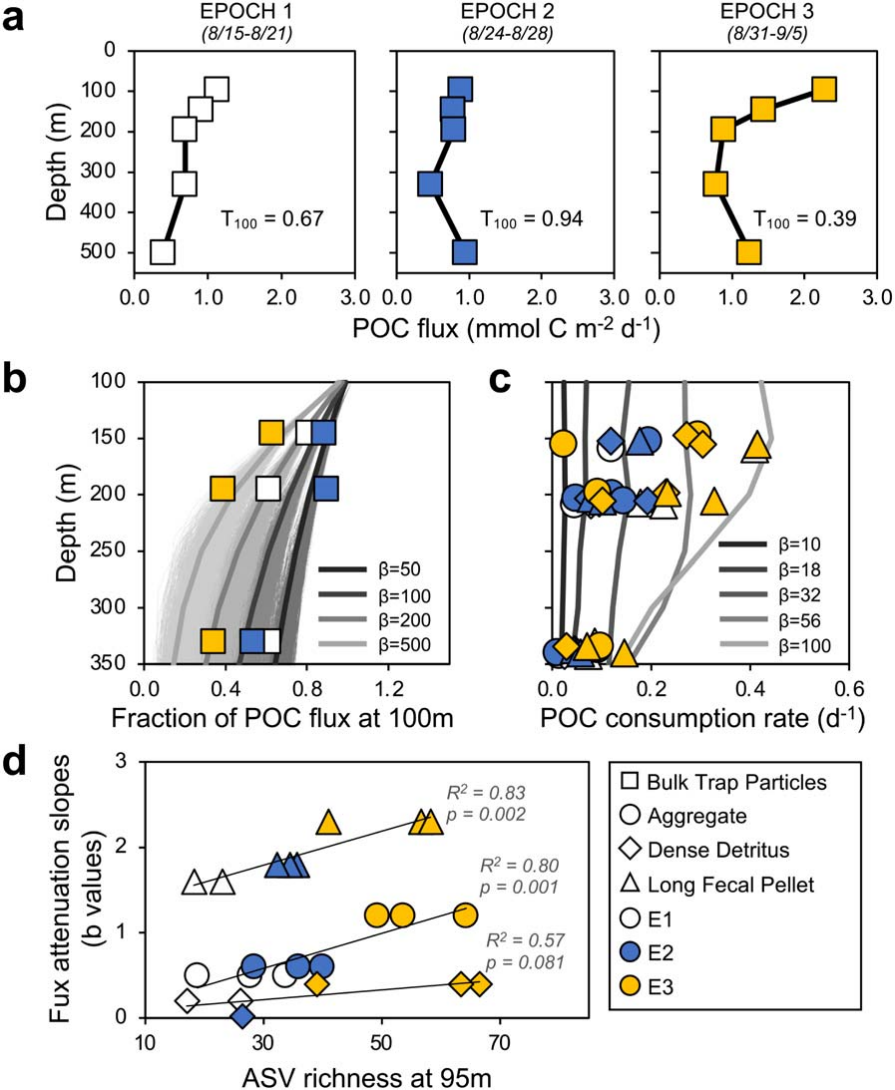
Dominant taxa on individual particles change with temporal changes in sinking flux attenuation; (A) particle flux and mean transfer efficiencies at 100 m (*T*_100_) as previously presented [33]; *T*_100_ = fraction of sinking carbon export transferred 100 m below the base of the euphotic zone; (B) temporal shifts in *T*_100_ are predicted by particle lability (ß = average particle lability (mmol C_POC_ mmol C_cell_^−1^ day^−1^) of all particles at formation depth of 100 m) in a mechanistic model of particle decomposition [8]; these modeled average temporal shifts are represented as lines from least to most labile for ß = 50, 100, 200, and 500 mmol C_POC_ mmol C_cell_^−1^ day^−1^; bold lines represent the average flux from each simulation (shaded lines represent *n* = 69 000 particles), and squares represent observed POC fluxes for E1, E2, and E3; (C) POC consumption rate (day^−1^) on individual particles is also predicted by modeled shifts in POC lability; model average POC consumption rates of individual particles are calculated for six discrete lability classes from 10 to 100 mmol C_POC_ mmol C_cell_^−1^ day^−1^; observational data of POC consumption rates of individual particles for each Epoch; (D) ASV richness of the microbial community associated with aggregates and long fecal pellets collected at 95 m was significantly and positively correlated (*P* <  .01) with flux attenuation slopes for those particle types; flux attenuation slope values were determined and previously reported in Durkin *et al*. [36]; “E” = Epoch.

Changes in individual particle community richness over time imply that microbial community diversity may influence the rate that sinking carbon is attenuated with depth (Fig. 4A), where a shift toward more diverse organic substrates may have supported a more diverse community (Fig. 4D) [32]. We hypothesize that the high flux attenuation and lower transfer efficiency (i.e. a lower ratio of POC flux at 200 m to the flux at 100 m; Fig. 4A) [33] in Epoch 3 were due to elevated production of more labile particulate organic matter and subsequent increased microbial remineralization between 95 and 145 m depth. A particle-based model that represents differential POC lability and variable particle-associated microbial community dynamics [8], using environmental conditions observed during the EXPORTS cruise, supports this hypothesis. We show that our observed temporal trends in sinking POC attenuation rates could be captured by shifts in POC lability (Fig. 4B) and changes in microbial consumption rates (Fig. 4C; symbols as observations and lines as model output). Additionally, we observed a significant positive correlation between POC flux attenuation and ASV richness for aggregates (*R*^2^ = 0.80, *P* =0 .001, *n* = 9) and long fecal pellets (*R*^2^ = 0.83, *P* = .002, *n* = 8; Fig. 4D), indicating that changes in POC lability favored shifts in microbial community composition. Greater ASV richness associated with increased attenuation during Epoch 3 could be attributed to wider metabolic capabilities from a greater number of taxa. Bulk trap-intercepted particles also had appreciably greater ASV richness during Epoch 3 at 95, 145, and 195 m as compared to earlier in the cruise (Supplementary Fig. 7), demonstrating that the temporal patterns captured in individual particles were also observed in other sinking particle sample types, though for shifts in a different microbial community composition (Fig. 1A). Slight offsets of POC flux observations from model at 330 m depths (i.e. deviation between observations in a given Epoch from model runs initialized at a single lability; Fig. 4B) could be reflective of processes not captured by the model (e.g. particle aggregation).

### Microbial community succession

**Figure 5:**
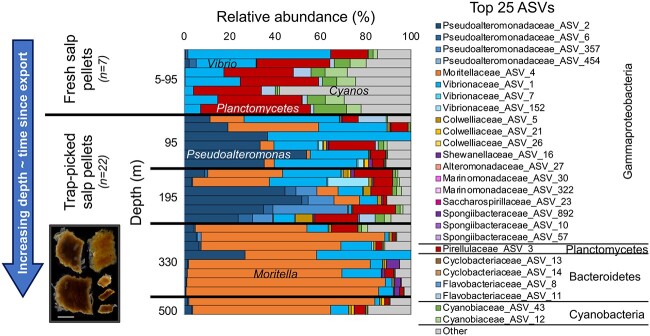
Microbial community succession patterns on sinking particles identified in situ on salp fecal pellets; relative abundances of the top 25 recurring ASVs shift as a function of depth moving from fresh salp pellets to individual sinking salp pellets from gel traps deployed at four depths, suggestive of community succession on particles as they sink through the water column; scale bar in image: 1000 μm.

Shifts in individual particle-associated communities by Epoch (Supplementary Figs 5, 7, and 8) are suggestive of successional trends, the process of which is hypothesized to play an important metabolic role by the community assembly on particles [13]. To examine potential succession patterns more specifically, we tracked the 25 most frequently observed ASVs associated with fecal pellets from salps, a gelatinous zooplankton that was transiently abundant during the expedition [36, 38]. Salp fecal pellets are large relative to those of other zooplankton, have a distinguishable square shape (Fig. 5), can retain a highly active bacterioplankton community [88], and played a disproportionally important role in POC export efficiency during the EXPORTS cruise when present in elevated abundances (up to 4 m^−3^) at Station Papa [39] and in other ocean regions [89].

Fresh salp pellets initially contained elevated relative abundances of *Vibrionaceae*, *Planctomycetes*, and *Cyanobiaceae* (combined relative abundance >80%; Fig. 5). These ASVs proportionally decreased as a function of depth in salp pellets from gel traps and were replaced first by *Pseudoaltermonadaceae* ASVs (up to 50% relative abundances), then by members of *Moritellaceae* (up to 80% relative abundance by 330 m). Assuming the majority of pellets were produced near the surface and using collection depth as a proxy for particle age, we interpret the loss of ASVs from fresh pellets and replacement by specific ASVs as community succession driven by particle colonization, grazing by flagellates, and detachment [76]. Although relatively elevated abundances of the predominant taxa are likely influenced by high copy numbers of the 16S rRNA gene per genome (e.g. 9–20 for the taxa highlighted here) [90] compared with typical average copy numbers for most bacteria (e.g. ~2) [91], the presented spatial and temporal ASV shifts could also be a measure of community turnover times. For example, using particle sinking rates of 500 m day^−1^ for particles 600 μm in ESD [92], mean copy numbers, and mean cell abundances for particle-attached bacteria (see Methods), we estimate that turnover times of 0.7–1.9 day^−1^ for *Moritellaceae* would have resembled growth rates commonly reported for this taxon [52, 54, 93].

**Figure 6:**
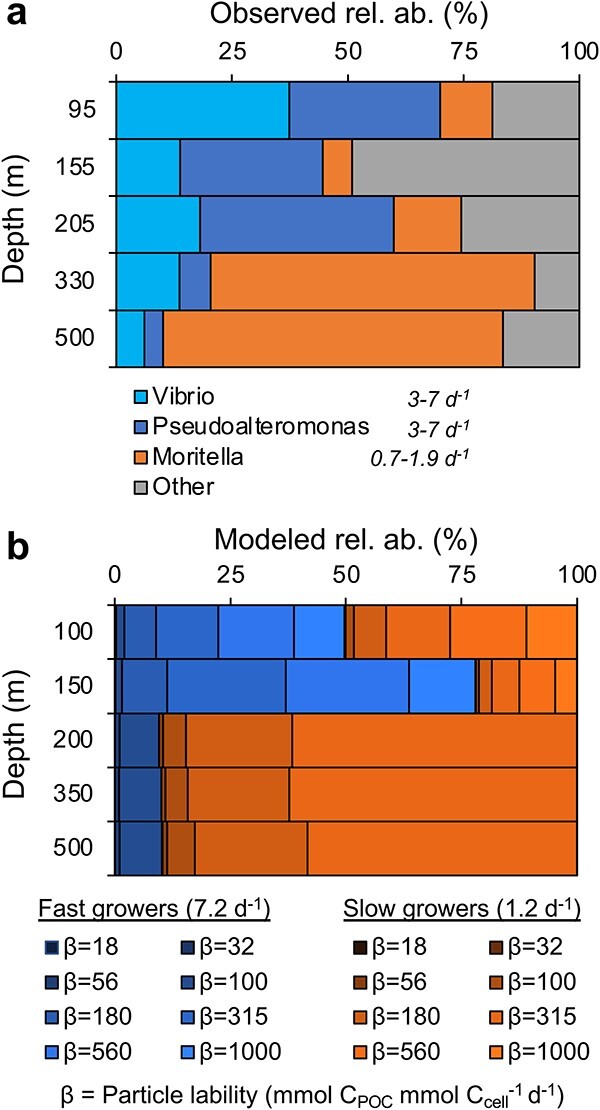
Microbial community succession on salp fecal pellets was similar to modeled community changes based on growth rates; (A) observed depth-based changes in dominant taxa on salp fecal pellets were similar to (B) the modeled changes in community growth rates, from taxa with faster growth rates (7.2 day^−1^) to slower growth rates (1.2 day^−1^); the different ß values in (B) refer to different particle labilities in units of mmol C_POC_ mmol C_cell_^−1^ day^−1^, where larger ß values refer to particles with higher lability; representing the particle labilities for fast and slow growers; the model community results are summarized from 1000 simulations of high average particle lability (ß = 500 mmol C_POC_ mmol C_cell_^−1^ day^−1^); in each simulation, we extract 161 particles belonging to particle size classes of 2000 and 4000 μm in diameter for this analysis to make it comparable to the size of salp pellet particles.

The effect of growth rate on microbial community succession on salp pellets is supported by a comparison of mean observed changes across all individual particle types (Fig. 6A) and modeled changes in community-based growth rate differences (Fig. 6B). The model output is reflective of a range of seeded particle types and a community with a range of enzyme kinetics. The model does not make assumptions about the relationship between growth rate and compound type [39]. As such, we find that the model community associated with particles at the shallowest equivalent trap depth was initially dominated by faster growing prokaryotes [52] with maximum growth rates of up to 7.2 day^−1^, and by 200 m, the community shifted to slower growers with growth rates of 1.2 day^−1^ (Fig. 6B). The observed community shift on individual salp fecal pellets from *Vibrio* and *Pseudoalteromonas*, which are typically fast-growing species [51, 94], to *Moritella* by 330 m (Figs 5 and 6A) is consistent with the model dynamics (Fig. 6B). The predicted shifts in microbial growth rate and observed shifts in dominant taxa (Fig. 6), may also be reflective of particle lability, where the fast-growing taxa that are no longer supported by labile substrates are replaced by slower growing microbes within particles. However, other potential influences on community succession, including pressure changes, preferential grazing, or competition, cannot be ruled out with the available data.

The specific successional patterns of the taxa associated with salp pellets that we observed with depth (Fig. 5) are supported by previous observations. For example, *Vibrionaceae* were detected in and on copepods near the Bermuda Atlantic Time-series Study site [95, 96], and the presence of *Vibrionaceae* on sinking particles was then used to implicate copepods as the primary source of sinking particles [20]. In another study, *Moritellaceae* and *Pseudoalteromonadaceae* were elevated in abundance in nonpoisoned traps compared with elevated *Vibrionaceae* in poisoned traps [97], suggesting that Vibrionaceae were present within freshly produced sinking particulate organic matter, but over time the microbial community shifted to other taxa. *Moritellaceae* are piezophilic and associated with particles found in the deep waters of the ocean (e.g. 4000 m), with metabolic characteristics allowing them to survive either on highly degraded particles or in high-pressure environments [6, 24]. Therefore, the variable presence of *Moritellaceae* in gel traps in the current study is suggestive of a colonization process on sinking particles.

**Figure 7:**
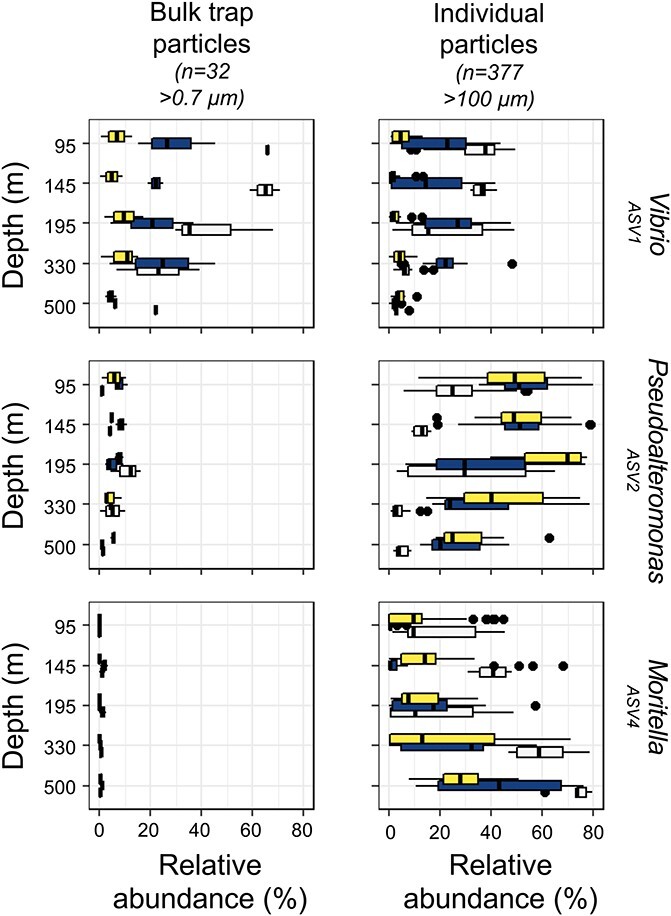
Dominant particle-associated taxa differ across sample type, depth, and time; ASV richness and relative abundances for the top three recurring ASVs for the individual particles differ by particle collection type (i.e. bulk particles from sediment traps vs. individual particles from gel traps), depth and time (i.e. “E” = Epoch); the line inside the box plots represents the median ASV richness, whiskers represent the minimum and maximum values excluding outliers, and the dots represent outliers (1.5 times the interquartile range).

Successional patterns for the “top 3” recurring ASVs on salp pellets were also observed among all isolated individual particle types as a function of depth and time (Fig. 7 and Supplementary Fig. 7B). These patterns were also detected in the broader community composition through an ordination analysis (Supplementary Fig. 8). For example, *Vibrionaceae* amplicon (ASV1 in Fig. 7) decreased with depth and time over the cruise in both the individual particle and bulk trap assemblage datasets. If *Vibrionaceae* are derived from zooplankton in the current study, their relative decrease and the increase by *Pseudoaltermonadaceae* (ASV2) in Epoch 3 (Fig. 7) suggest a more rapid succession associated with increased downward POC flux and lability (Fig. 4). The higher rates of surface NPP and BCD (Supplementary Fig. 3) and an increased lability inferred by sinking flux attenuation (Fig. 4) could have supported a greater number of taxa as well as a relative increase in *Pseudoalteromonas* on individual particles. The temporal-based shifts in the particle-associated microbial communities could have also played a role in the increased sinking particle remineralization, as suggested by reduced transfer efficiency in Epoch 3 [33].

Our results add to a growing understanding of the connection between surface primary production and subsurface microbial community dynamics (Fig. 4C). A relative increase in taxa related to *Moritella* and *Colwellia* was observed on particles collected near the seafloor (4000 m) during diatom blooms and periods of elevated flux outside of Monterey Bay [98], suggesting that high flux events led to more efficient transport of surface produced material and their associated microbial communities. At station ALOHA in the central North Pacific (4000 m), there were enhanced sediment trap contributions from copiotrophic *Alteromonadaceae*, *Flavobacteriaceae*, *Rhodobacterales*, *Oceanospirillale*s, and *Vibrionaceae* associated with summertime export pulses fueled by increases in the biomass and productivity of nitrogen-fixing cyanobacteria and diatoms [24]. Also, at Station ALOHA, increased particle export at 175 m was associated with enhanced growth response by members of *Pseudoaltermonadales* and *Vibrionales* grown on particles, demonstrating that an increase in surface NPP can influence particle-associated bacterial growth response [99]. Altogether, surface-enhanced productivity could provide more labile organic resources (Fig. 4B), which then could have favored relative increases in the *Pseudoaltermonadaceae* later in our study period (Fig. 7).

### Particle-attached bacterial metabolism

The comparison of our 16S rRNA gene amplicon data among sample methods (Fig. 1), depth, and time patterns suggested that environmental factors controlling the richness of particle-associated communities at the shallowest depths influenced carbon flux attenuation rates into the mesopelagic zone (Fig. 4C). The microbial communities on these attenuating particles also exhibited consistent succession dynamics among key taxa with depth (Figs 5–7); thus, we infer that these taxa were influenced by environmental variability over time and depth. To assess whether specialized metabolic capabilities responsible for this succession could be detected in the genomes of the predominant taxa identified on individual particles from gel traps, we conducted metagenomic sequencing and assembly on a subset of pooled individual particles collected from specific depths and times during the cruise. Metagenome-assembled genomes (MAGs) were successfully generated for the three dominant ASVs recovered in the amplicon dataset (*Vibrio*, *Pseudoalteromonas*, and *Moritella*), though the MAG for the *Vibrionaceae* representative (corresponding to ASV1) had relatively low completeness (57.2%; Supplementary Table 2). The community composition was highly similar between 16S rRNA gene amplicons and the relative abundance of the *recA* gene in the unassembled metagenome data (Supplementary Fig. 9), providing evidence that the two sequencing and postprocessing methods resulted in comparable data.

An ASV related to *Vibrio* was in high relative abundance in fresh salp fecal pellets and decreased with depth in both salp pellets (Figs 5 and 6) and in other isolated individual particle types (Fig. 7), suggesting that *Vibrio* may be associated with salps and other abundant zooplankton at our study site (e.g. amphipods) [38]. Consistent with this, the *Vibrio* MAG contained genes for alginate and chitin degradation, as well as multiple genes involved in anaerobic respiration (Supplementary Table 3). *Vibrio* species are associated with the guts, carapaces, and fecal pellets of copepods [96, 100-103], which are well known to obtain carbon and nitrogen energy sources from chitin [104, 105]. Similarly, we hypothesize that the elevated detection of *Vibrio* in particles is associated with recent zooplankton production (Fig. 5).

*Pseudoalteromonas* and *Moritella*, which increased in relative abundance with depth, were enriched in motility and translation KO modules, particularly in the synthesis of peptides in cell wall biosynthesis (Supplementary Fig. 10). An increase in cell wall and translation genes in *Pseudoalteromonas* and *Moritella* could reflect an elevated capability to resist antagonistic strategies such as secretion of harmful enzymes, which, when coupled with antibacterial and type VI secretion genes, could allow these taxa to increase in abundance (Supplementary Table 3). The ability to resist antagonistic strategies is further supported in *Pseudoalteromonas*, with its elevated defense and certain signal transduction clusters of orthologous groups (COGs) of proteins. For example, *Pseudoalteromonas* has several beta-lactamase penicillin-binding proteins and other antibiotic resistance *AmpC* type genes, neither of which were detected in *Vibrio* or *Moritella* (Supplementary Table 3). This, coupled with amino acid transport COGs that included several peptidases unique to *Pseudoalteromonas*, could have led to its replacement of *Vibrio* on particles as they sank. Enhanced amino acid transport could have also allowed *Pseudoalteromonas* to become relatively more abundant during Epoch 3, given that amino acids were elevated in recently produced dissolved organic matter in surface waters [32]. This suggests that the quality of exported material influenced the responses of heterotrophic bacterial communities. The modeled shift toward more labile POC during Epoch 3 (Fig. 4B), coupled with elevated *Pseudoalteromonas* abundances on individual particles (Fig. 7), also suggests that *Pseudoalteromonas* played a role in enhancing the attenuation of sinking particles.

Anaerobic nitrogen-cycling pathways were detected in *Moritella* and *Vibrio* in addition to genes associated with urea processing, suggesting that nitrogen metabolism was important and that oxygen levels inside the particles could have been low. For example, the *Vibrio* and *Moritella* MAGs contained *nrfA* and *nrfD* genes, responsible for dissimilatory nitrite reduction to ammonium, and *nap* genes, responsible for nitrate reductase. The *Moritella* MAG contained genes for two intermediate steps in the respiratory denitrification pathway, nitric oxide reductase and nitrous oxide reductase (Supplementary Table 3). The finding that *Moritella* was either undetectable or had very low abundances in the bulk sinking particle assemblage, 5.0 μm filters, and fresh fecal pellets, and that it increased with depth on the individual particles (>100 μm in ESD) from gel traps (Figs 5 and 7), implies that this taxon was associated with less labile particulate organic matter that allowed it to become more prevalent under anoxic conditions on or within larger individual particle micro-environments. Anaerobic metabolism pathways associated with particles were previously detected at 4000 m depth near station ALOHA and were hypothesized to either reflect co-occurring aerobic and anaerobic pathways on particles or a symbiotic association of facultative anaerobic bacteria within the guts of mid-water protists or larger animals [106]. Models suggest that denitrification in particulate matter may be responsible for nitrogen loss and nitrous oxide production, even in regions outside oceanic oxygen-deficient zones such as the subarctic North Pacific [107]. Our results support this model prediction at Station Papa, where water column dissolved oxygen levels were as low as 35 μM at 500 m [29], not low enough to have inhibited oxygen diffusion into the particles. Instead, the interior microenvironment of these larger (>100 μm ESD) individual particles likely reached anoxic conditions [108].

## Conclusions

The amount of particulate organic carbon transported to the deep ocean at any given place and time is not a simple linear function of primary production in the overlying surface waters [109], yet it is a critical component in understanding the ocean’s role in Earth’s climate system. Mechanistic models that include particle-attached microbial growth rate dynamics and differences in particle lability will improve the predictive capacity of this biological pump. This work supports the need to have dynamic rates of particulate organic matter (POM) degradation in large-scale biogeochemical models; simply using a single rate constant for POM attenuation may not accurately capture environmental processes, as presented here.

We present environmental evidence of successional patterns in bacterial communities attached to individual particles during a 3-week occupation of the North Pacific’s Ocean Station Papa. Community patterns on individual particles differed from those observed using other particle-associated methods, including bulk particles from sediment traps, fresh fecal pellets, MSCs, and 5.0 μm filters. The depth- and temporally based patterns observed between fresh fecal pellets and individual particles collected in gel traps suggest that environmental influences dominated the successional patterns from *Vibrio* to *Pseudoalteromonas* to *Moritella* with depth (i.e. time). Yet, we also observed a potential relationship between the initial community at particle formation and the eventual community assembled at depth, suggesting that historical contingency or priority effects (e.g. [110]) could be influencing microbial dynamics on particles. Furthermore, modeled growth rate differences and measured metabolic potentials for those three taxa provide a physiological basis for these successional patterns. Combining data similar to those presented here with particle-specific bacterial cell abundances, the 16S rRNA gene sequencing of individual particles could provide the field data necessary to test current models predicting the influence of particle-attached bacteria on flux attenuation [8]. In other words, models will require further environmental and microbial dynamic context to accurately predict influences on particle attenuation. The temporal and spatial patterns observed here suggest that continued study of community succession patterns in other environments will further our understanding of the processes influencing sinking flux and particle-associated community dynamics.

## Supplementary Material

EXPORTS_NP_ISME_SUPPLEMENT_Nov_2023_wrad010

Top_ASVs_from_Particles_Extended_Data_Table_1_wrad010

## Data Availability

Hydrographic, sinking flux, and sequencing data can be found at the NASA-supported https://seabass.gsfc.nasa.gov/experiment/EXPORTS. 16S rRNA gene sequences can also be found under NCBI BioProject accession number PRJNA910148, and MAG assemblies have been submitted (available under the same accession number upon dataset publication).
