# Supplementary material for: Direct observations of microbial community succession on sinking marine particles
Source: ISME J. 2024 Jan 10;18(1):wrad010. doi: 10.1093/ismejo/wrad010 (PMC10811735; doi:10.1093/ismejo/wrad010)
Supplement: EXPORTS_NP_ISME_SUPPLEMENT_Nov_2023_wrad010 [file exports_np_isme_supplement_nov_2023_wrad010.pdf]

Supplemental Information

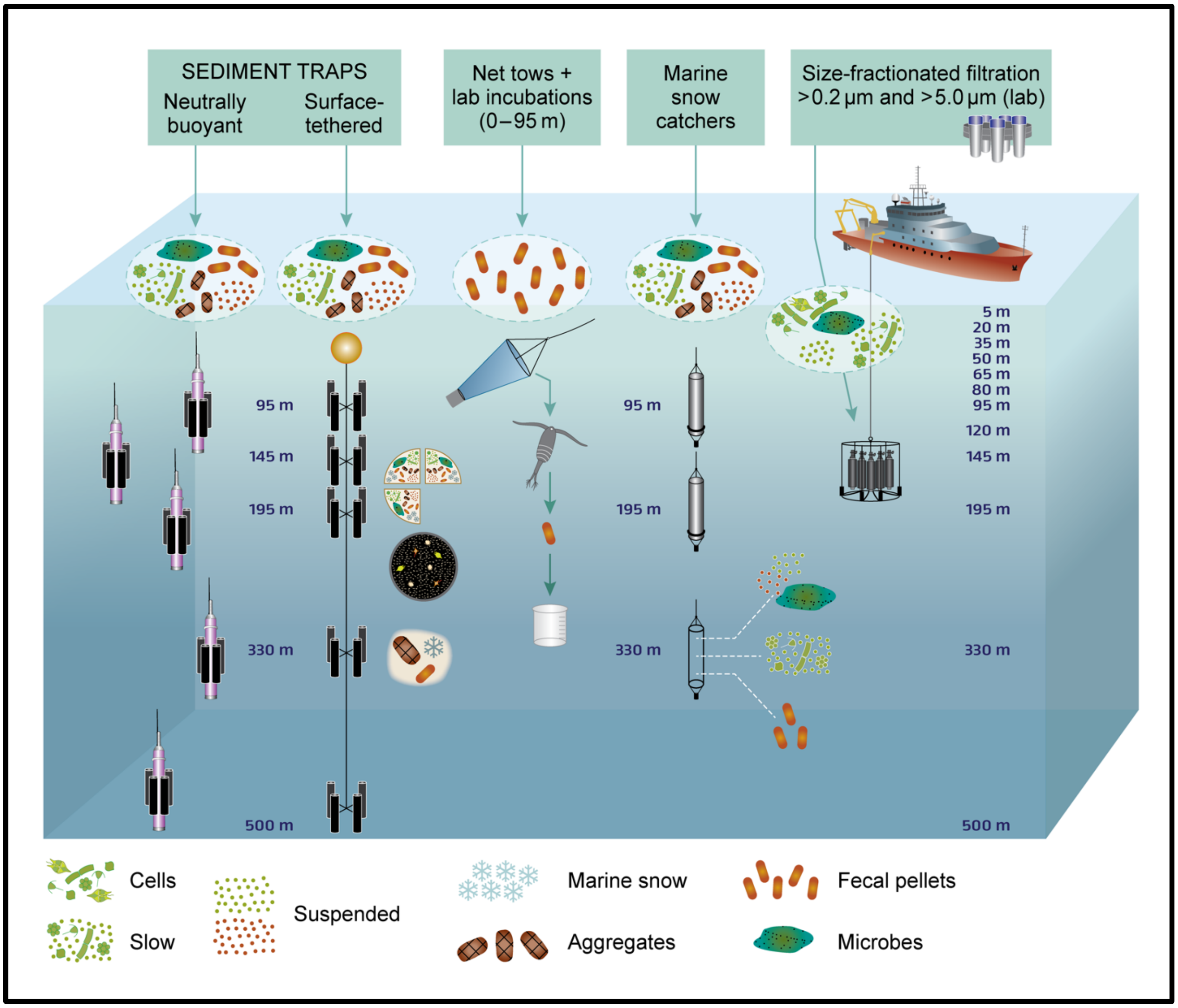

Supplementary Fig. 1. | Sampling methods employed during the North Pacific EXPORTS cruise. Sampling methods included the collection of samples representing individual particles from gels and the bulk assemblage from sediment traps, net tows followed by shipboard incubations of zooplankton, marine snow catchers (MSCs) and size-fractionated filtration from water collected using Niskin bottles. Noted are the range of particle types sampled by each method and the depths from which the samples were collected. Two sediment trap types were deployed (neutrally buoyant and surface-tethered), and individual particles from gels and bulk particle samples were collected from both types of traps. Net tows collected samples over 0 to 95 m depths. Samples from the MSCs were divided into non-sinking, slowly-sinking and fast-sinking fractions. Size-fractionation was performed in series for 95 to 500 m depths, where 5.0 μm filtrate was filtered through 0.2 μm. Samples collected onto 0.2 μm without pre-filtration were also collected separately and found to have similar communities to the 0.2 to 5.0 μm filter samples collected by size-fractionation.

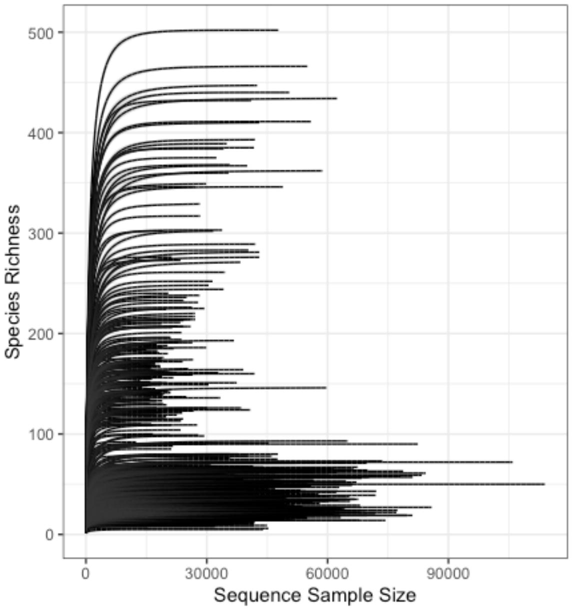

Supplementary Fig. 2. **| Rarefaction curves for samples analyzed in manuscript.** 578 samples were analyzed at an average read depth of 36,629 ± 16,565 reads per sample. All samples reached a maximum species richness by 5,000 reads.

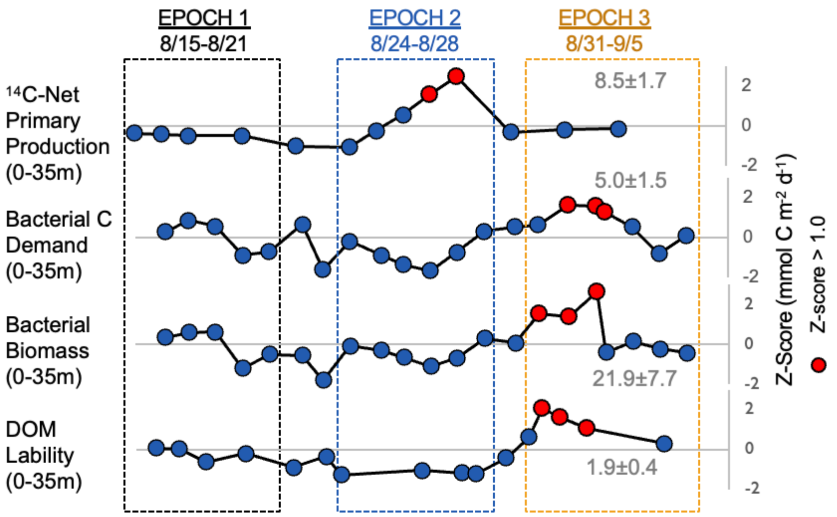

Supplementary Fig. 3. **| Temporal changes in surface production bacterial dynamics and DOM lability.** Mixed layer ^{14}C-net primary production, bacterioplankton carbon demand (BCD), bacterioplankton biomass (BB) and dissolved organic matter (DOM) lability exhibited significant increases (<1.0 standard deviation from the mean; denoted by red circles) during in the 3rd EPOCH of the 24-day station occupation. Data for NPP, BCD, BB and DOM lability as published in Stephens et al. (2023) [1]. The DOM lability values are derived from an amino acid-based indicator of degradation. Noted for each measurement are the cruise mean ± standard deviation in grey text within each subplot, and the depths for which the data are presented in parentheses on the lefthand axis.

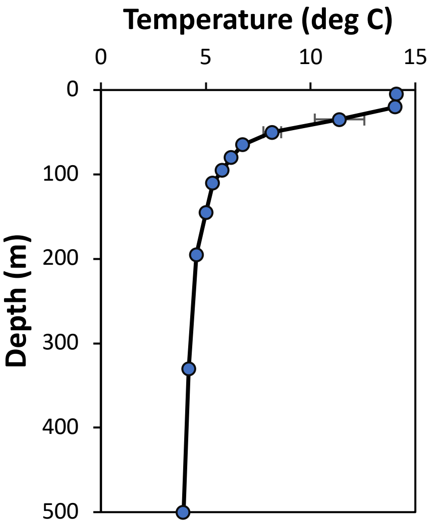

Supplementary Fig. 4. **| Mean temperature profile collected from EXPORTS cruise RR1813 in the North Pacific.** Error bars represent the standard deviations for 24 profiles, from August 15th to September 4th, 2018.

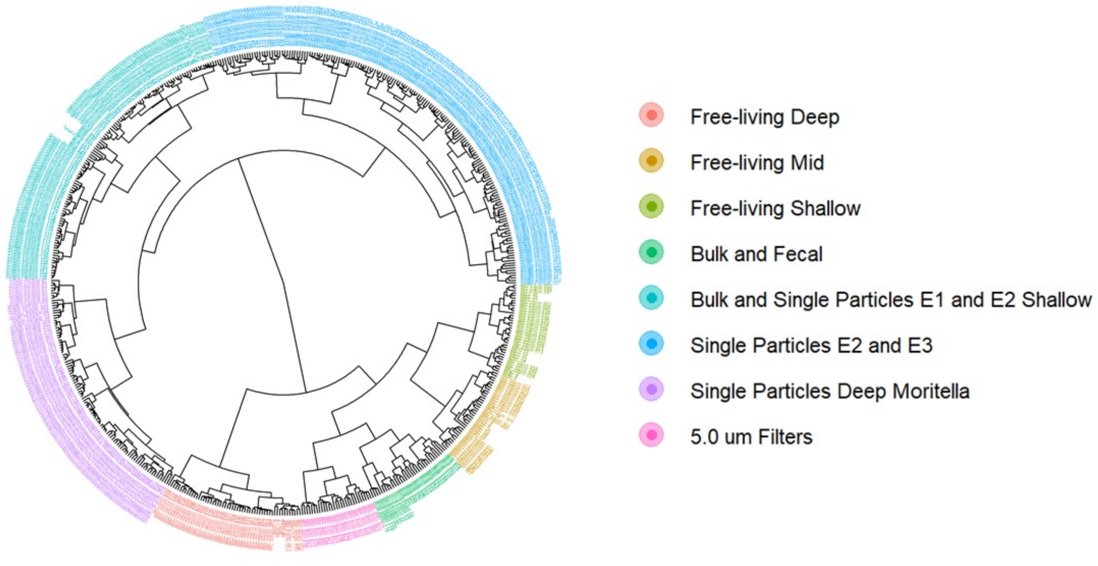

Supplementary Fig. 5. **| Clustering of amplicon-based communities by sample type.** Bray-Curtis-based clustering (k = 8) among sample collection types presented here.

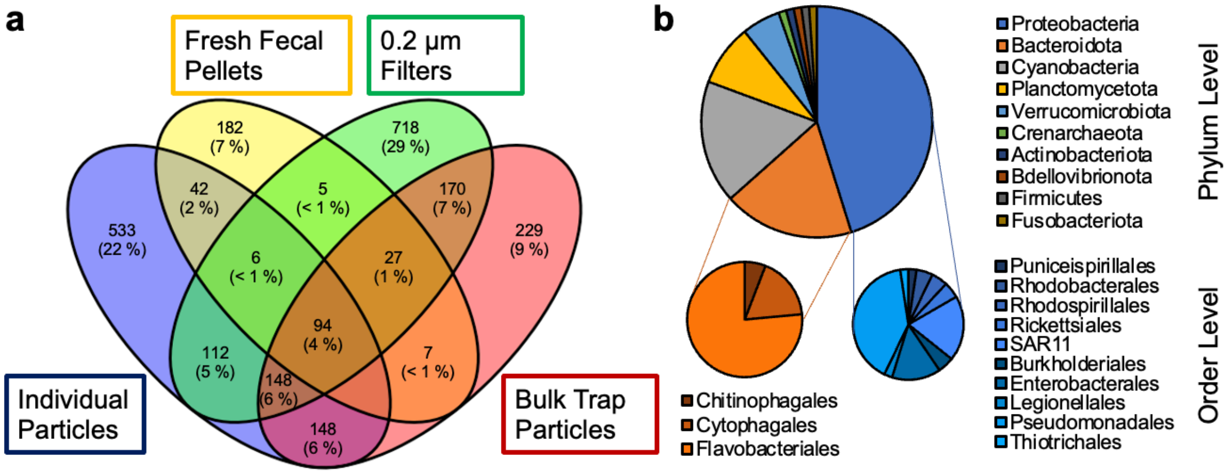

Supplementary Fig. 6. | Shared taxa by sample type. (a) Venn diagram of shared amplicon sequence variants (ASVs) across four of the sample collection types presented here. (b) Of the 94 shared ASVs, 45 % were *Proteobacteria*, 18 % were *Bacteroidota* and 17 % were *Cyanobacteria*. Of the shared *Proteobacteria* ASVs, 40 % were *Pseudomondales*, 19 % were SAR11 and 14 % were *Enterobacterales*. And of the shared *Bacteroidota* ASVs, 76 % were *Flavobacteriales* and 18 % were *Cytophagales*.

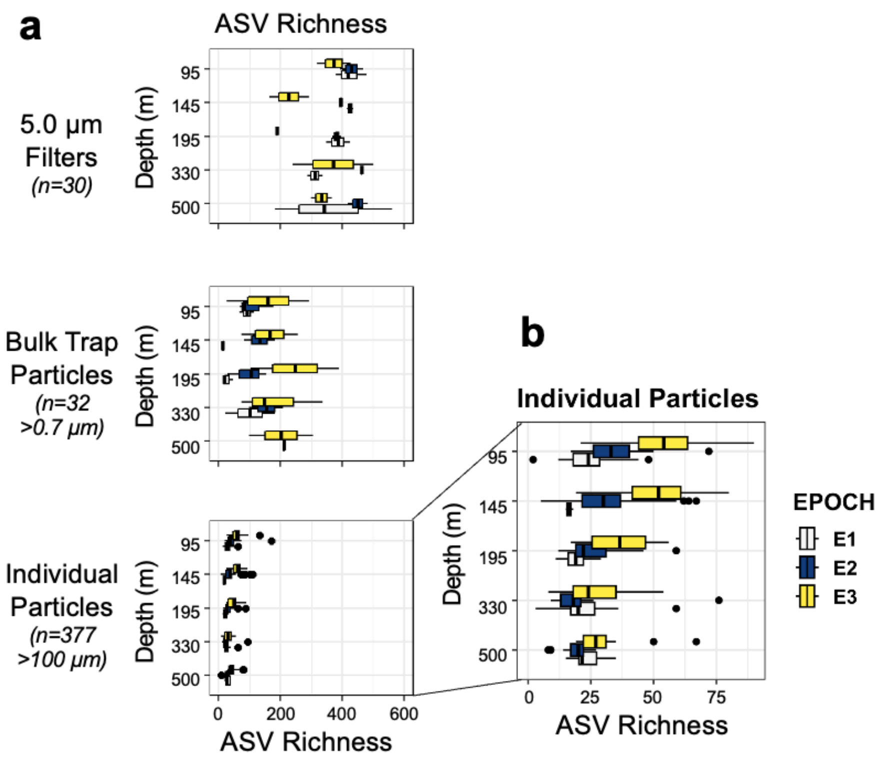

Supplementary Fig. 7. | ASV richness differed by EPOCH and by sample type. (a) Amplicon sequence variant (ASV) richness differed by EPOCH for different particle collection types (i.e., 5.0 µm filters, bulk particles, individual particles). (b) Individual particle depth trends with a constrained x-axis highlighting the elevated ASV Richness during Epoch 3. Blue boxes = Epoch 1, white boxes = Epoch 2 and yellow boxes = Epoch 3. The line inside the box plots represents the median ASV richness, whiskers represent the minimum and maximum values excluding outliers and the dots represent outliers (1.5 times the interquartile range).

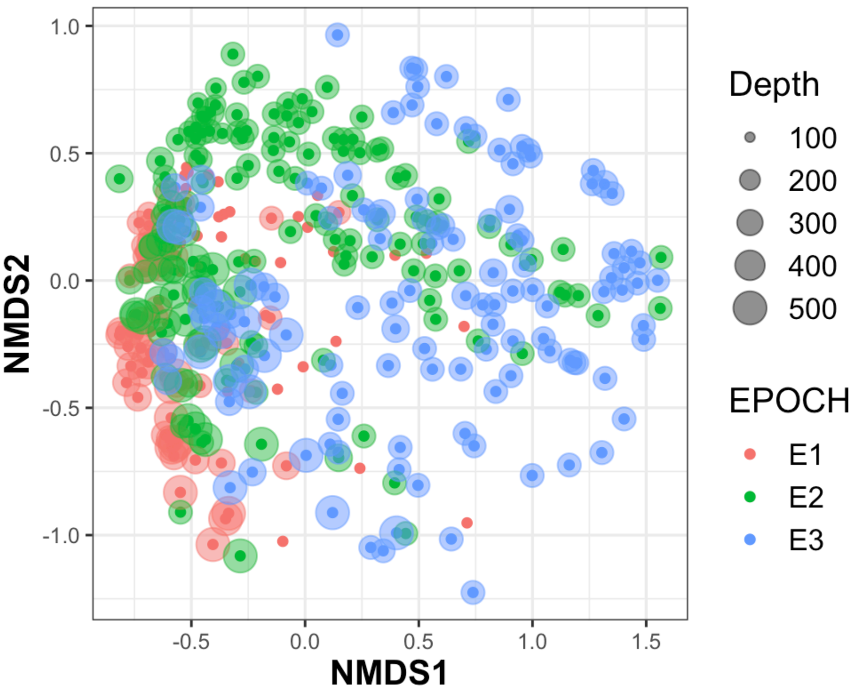

Supplementary Fig. 8. **| Individual particle communities differed by Epoch.** Non-parametric multidimensional scaling ordination of 16S rRNA gene amplicon sequence variants for individual particles exhibited modest differences based on time (i.e., Epoch). “E” = Epoch.

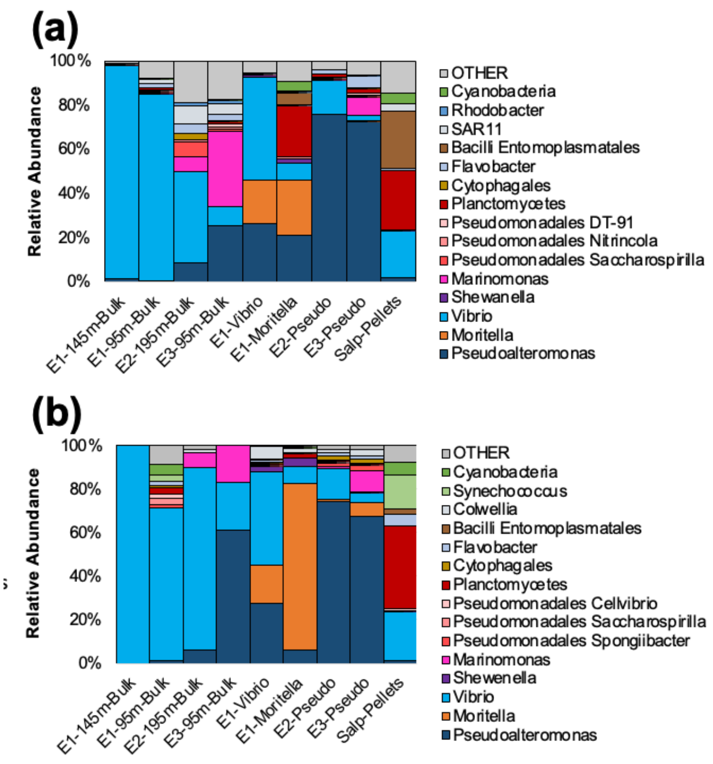

Supplementary Fig. 9. | Communities characterized by different sequencing methods were similar. Comparison of relative abundances for taxa determined by (a) metagenomic sequencing (Illumina NextSeq) and based on the *recA* gene and genome-based taxonomy database (GTDB) and (b) 16S rRNA gene amplicon sequencing, the DADA2 pipeline and Silva database v138.1.

a

		Vibrio	*Pseudoalteromonas*	*Moritella*
Genome size & Est. Genome (bp)		2,266,700<br>3,959,993	5,484,496<br>5,545,496	4,590,147<br>4,926,108
GC (%)		44.45	39.41	40.50
Completeness (%)		57.20	98.90	93.20
COG Categories		Carbohyd. Transport (G)<br>Nucleotide Transport (F)<br>Transcription (K)<br>Intracellular Trafficking (U)	Signal Transduction (T)<br>Amino Acid Transport (E)<br>Defense (V)<br>Cell Wall Biogenesis (M)	Energy Production (C)<br>Coenzyme Transport (H)<br>Lipid Transport (I)<br>Inorganic Ion Transp. (P)
Sugar-Acid Preference	*Negative=acid preference*	-0.45	-0.51	-0.45
KO Module and Categories	*Antibacterial & Type VI Secretion*	✓	✓	✓
	Cellulose Degradation	✓	✓	✓
	NO3, NO2, NO, N2O reduction	✓		✓
	Urea Processing	✓		✓
	Chitinases	✓	✓	✓
	Motility	✓	✓	✓
CAZymes	*Glucose*	✓	✓	✓
	Galactose	✓	✓	✓
	Terminal Sugars	✓	✓	✓
	Alginate	✓		✓

b

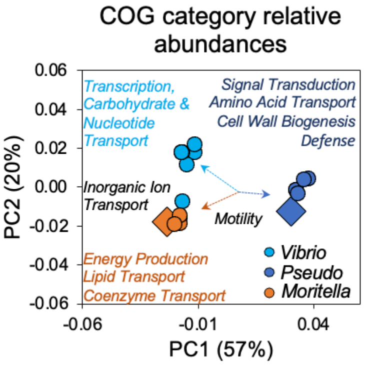

Supplementary Fig. 10. | Dominant taxa on individual particles differ in their metabolism. (a). The relative contributions and/or presence of clusters of orthologous groups (COGs) of protein categories, KEGG orthology (KO) modules and carbohydrate-active enzyme (CAZyme) pathways differed in metagenome assembled genomes (MAGs) of *Pseudoalteromonas*, *Moritella* and *Vibrio.* The sugar-acid preference was determined based on the number of sugars and acids that each organism can grow on (Gralka et al., 2023) [2]. (b) Metabolic capability from the three dominant taxa differs in ordination space based on the relative contributions of different COGs in each taxon. MAGs obtained from individual particles (large diamonds) were supplemented with the sequenced genomes from the Integrated Microbial Genomes and Microbiomes database (small circles) with >99.5 % identity similarity.

Supplementary Table 2. | Mean relative abundances of clusters of orthologous genes (COG) categories for MAGs and publicly available genomes.

Function ID	Function Name	*Moritella*	*Vibrio*	*Pseudo-alteromonas*
C	*Energy production and conversion*	6%	6%	5%
D	*Cell cycle control, cell division, chromosome partitioning*	1%	1%	1%
E	*Amino acid transport and metabolism*	8%	9%	8%
F	*Nucleotide transport and metabolism*	3%	3%	2%
G	*Carbohydrate transport and metabolism*	4%	6%	4%
H	*Coenzyme transport and metabolism*	5%	5%	4%
I	*Lipid transport and metabolism*	4%	3%	4%
J	*Translation, ribosomal structure and biogenesis*	7%	7%	7%
K	*Transcription*	7%	8%	7%
L	*Replication, recombination and repair*	4%	4%	4%
M	*Cell wall/membrane/envelope biogenesis*	6%	6%	7%
N	*Cell motility*	4%	3%	4%
O	*Posttranslational modification, protein turnover, chaperones*	5%	5%	5%
P	*Inorganic ion transport and metabolism*	5%	5%	5%
Q	*Secondary metabolites biosynthesis, transport and catabolism*	2%	2%	2%
T	*Signal transduction mechanisms*	7%	7%	9%
U	*Intracellular trafficking, secretion, and vesicular transport*	3%	3%	2%
V	*Defense mechanisms*	2%	3%	3%

Supplemental References

1. Stephens BM, Fox J, Liu S, Halsey KH, Nicholson DP, Traylor S, et al. Influence of amino acids on bacterioplankton production, biomass and community composition at Ocean Station Papa in the subarctic Pacific. *Elementa* 2023; **11**.
2. Gralka M, Pollak S, Cordero OX. Genome content predicts the carbon catabolic preferences of heterotrophic bacteria. *Nature Microbiology 2023 8:10* 2023; **8**: 1799–1808.
